# Cardiac Amyloidosis with Normal Wall Thickness: Prevalence, Clinical Characteristics and Outcome in a Retrospective Analysis

**DOI:** 10.3390/biomedicines10071765

**Published:** 2022-07-21

**Authors:** Daniella Nagy, Katalin Révész, Gergely Peskó, Gergely Varga, Laura Horváth, Péter Farkas, András Dávid Tóth, Róbert Sepp, Hajnalka Vágó, Anikó Ilona Nagy, Tamás Masszi, Zoltán Pozsonyi

**Affiliations:** 1 Department of Internal Medicine and Haematology, Semmelweis University, H-1088 Budapest, Hungary; nagydana27@gmail.com (D.N.); revesz.katalin@med.semmelweis-univ.hu (K.R.); pesko.gergely@med.semmelweis-univ.hu (G.P.); vargager@gmail.com (G.V.); horlau@gmail.com (L.H.); lupusetuxor@gmail.com (P.F.); toth.andras1@med.semmelweis-univ.hu (A.D.T.); masszi.tamas@med.semmelweis-univ.hu (T.M.); 2 Division of Non-Invasive Cardiology, Department of Internal Medicine, University of Szeged, H-6725 Szeged, Hungary; sepprobert@gmail.com; 3 Heart and Vascular Center, Semmelweis University, H-1122 Budapest, Hungary; vagoha@gmail.com (H.V.); anychophora@gmail.com (A.I.N.); 4 Department of Medicine, Karolinska Institute, 17177 Stockholm, Sweden

**Keywords:** cardiac amyloidosis, AL amyloidosis, ATTR amyloidosis, left ventricular wall thickness, cardiac imaging

## Abstract

Background: Cardiac amyloidosis (CA) is a rare, progressive, infiltrative cardiac disease. Light chain (AL) and transthyretin (ATTR) amyloidosis are in the background in almost all cases. New, easily available diagnostic tools and recently introduced novel therapies for both types of CA put this disease into the field of interest. Increased left ventricular wall thickness (IWT) detected by echocardiography is generally thought to be a necessary part of the diagnosis. We aimed to determine the proportion of CA patients without IWT, and to define the clinical characteristics of this cohort. Methods: In an academic tertiary center for CA, we identified patients diagnosed and treated for CA between January 2009 and February 2022. In a retrospective analysis we defined the proportion of patients with (≥12 mm) and without (<12 mm) IWT, and described their clinical features. Results: We identified 98 patients suitable for the analysis. In total, 70 had AL and 27 ATTR CA; 89 patients had CA with IWT and 9 patients (9%) had CA without IWT. All non-IWT patients had AL type CA. Both group of patients had clinically significant disease, which is supported by the relevant elevation in cardiac biomarker levels. There was no difference between the outcome of the two groups. Conclusion: Patients without IWT form a relevant subgroup among those with CA. Our results suggest that diagnostic algorithms and criteria should take these individuals into consideration, and, therefore, give them access to effective treatments.

## 1. Introduction

Cardiac amyloidosis (CA) is a progressive, infiltrative disease, causing HF and often has poor prognosis. The vast majority of the patients have either light chain (AL) or transthyretin (ATTR) systemic amyloidosis in the background. Cheap and simple diagnostic tools and novel therapies are available for both types [1], which is why CA has gained so much interest in recent years. Left ventricular wall thickening is generally thought to be mandatory for the diagnosis, and is part of prior diagnostic criteria [2,3]. However, AL type CA can be also diagnosed without the presence of wall thickening, and without typical imaging features according to other diagnostic criteria [4,5], as published recently. Expert recommendations allow diagnosis of AL type CA in the presence of a positive extracardiac biopsy and increased cardiac biomarkers, when all other causes of biomarker elevation can be excluded [4,5]. Increased left ventricular wall thickness was proposed as the most important sign for the clinical suspicion and clinical diagnosis of CA in the latest position statement on CA, published by the Working Group on Myocardial and Pericardial Diseases of the European Society of Cardiology [6]. According to this latest document, irrespective of a positive extracardiac biopsy, when IWT is lacking on echocardiography, the diagnosis of CA can only be confirmed if an endomyocardial biopsy demonstrates amyloid deposits or cardiac magnetic resonance imaging (CMR) shows typical signs of CA. The proportion, clinical features and outcome of “non-hypertrophic” CA patients remains unknown, although it would be relevant, as these patients may raise less suspicion for the disease and, therefore, may have longer diagnostic delay and less chance for effective therapy. In this retrospective trial we aimed to define the proportion and clinical characteristics of CA patients with normal (less than 12 mm) left ventricular wall thickness and to compare the characteristics and prognosis of these patients to those who had IWT and CA.

## 2. Material and Methods

### 2.1. Patients

All consecutive patients diagnosed and treated with CA between January 2009 and February 2022 at the Department of Internal Medicine and Haematology, Semmelweis University were retrospectively enrolled. Our department is a tertiary center for CA in Hungary. In our analysis, we defined CA according to the expert consensus recommendation on the imaging and diagnosis of CA, endorsed by eight societies and associations involved in this topic from the USA and Europe [4,5]. The study was conducted according to the guidelines of the Declaration of Helsinki and was approved by Ethics Committee of University of Szeged (165/2016-SZTE).

CA patients were divided into two groups, according to the presence of IWT. The left ventricular wall thickness was in the center of our interest; therefore, we excluded those patients from the analysis who also had other conditions resulting in IWT, such as significant aortic valve stenosis, prosthetic valve implantation or uncontrolled hypertension in the medical history. For the sake of the definitive diagnosis of CA, and to avoid misdiagnosis of CA based on N-terminal pro B-type natriuretic peptide (NTproBNP) elevation due to other causes than CA, we excluded patients from the non-IWT group, who had atrial fibrillation with uncontrolled ventricular response, or severe renal failure with concomitant fluid retention.

### 2.2. Echocardiography

Echocardiography was performed in accordance with current guidelines, using two machines: between 2009 and 2019 a Philips iE33 system, equipped with a S5-1 transducer, and, since 2019, a Philips EPIQ 7C system (both Philips, Amsterdam, The Netherlands), equipped with a X5-1 transducer. Left ventricular wall thickness was measured on 2D B-mode end-diastolic images. Wall thickness was defined as the average of the septal and posterior wall thickness. For strain analysis, we used QLab 10.5 software, (Philips, Amsterdam, The Netherlands). For the analysis of the regional strain differences (to find the so called apical sparing or “cherry on top” sign), we followed the method originally described by Phelan et al. [7], and used the following equation: relative apical LS (longitudinal strain) = average apical LS/(average basal LS + average mid LS). All measurements were performed by a single experienced echocardiographer.

### 2.3. Statistics

Since most of the variables exhibited skewed distributions, the descriptive statistics are presented as medians with interquartile ranges (IQR), or as percentages. The strength of the associations was calculated with the nonparametric Mann–Whitney test or the chi-square test, as appropriate. To compare the mortality of the IWT and non-IWT groups, we draw Kaplan–Meier curves to demonstrate the survival and used a Log–Rank test to study the possible differences between the groups. Intra-observer reproducibility of LV wall thickness measurements was tested using the intra-class correlation coefficient (ICC) in 15 randomly selected patients, with an at least two-week long interval between the two measurements.

For the statistical analysis we used Statistica Software, v13, StatSoft Inc, Tulsa, OK, USA.

## 3. Results

### 3.1. Patient Population

Between January 2009 and February 2022, we identified 100 patients treated for CA at our department. Two patients also had severe aortic valve stenosis, and were excluded from the analysis. Reproducibility measures were good for echocardiographic LV myocardial thickness assessment. The intra-observer intra-class correlation coefficient (ICC) for wall thickness measurement was 0.924 (0.933 for septal wall and 0.919 for posterior wall thickness). In Table 1, we summarized the most important echocardiographic, laboratory and clinical parameters of the patients We also compared these parameters between patients with and without IWT. 71% of our CA patients had AL, 27% had ATTR and one had AA CA.

For the diagnosis of CA, we used different imaging methods. Cardiac MRI was performed in 60 cases where 50 were positive for CA when only the presence of typical late gadolinium enhancement (LGE) was considered as sign of CA. The ten patients without a positive CMR did not get gadolinium based contrast material due to contraindications, had atrial fibrillation causing technical difficulties, did not complete the exam due to shortness of breath, or only mapping results suggested CA. Pyrophosphate (PYP) isotope scan was performed in 26 cases, (score was 0 in one case; 1 in 6 cases and 2 or 3 in 19 cases). The final diagnosis was ATTR in 19 cases among those who had a PYP scan. We analyzed left ventricular regional strain differences after the year of 2015, when echocardiographic images were suitable, and the results may have helped the diagnosis. In 22 cases, the strain analysis was performed, and relative apical sparing was greater than 1 in 9 CA cases.

The available specific treatments have changed in the time period when these patients were diagnosed, therefore their treatments were diverse. For AL patients, after the diagnosis of CA, the first line medical treatment was generally a combination of cyclophosphamide-bortezomib-dexamethasone (33 cases). Other regimes were also used: bortezomib-dexamethasone (10 cases), melphalan-prednisolone-bortezomib (7 cases), bortezomibe-thalidomide-dexamethasone (7 cases), bortezomib-lenalidomide-dexamethasone (2 cases), thalidomide alone (2 cases), melphalane-thalidomide (one case), thalidomide-dexamethasone (one case), cyclophosphamide-thalidomide-dexamethasone (2 cases), melphalan-prednisolone (one case) and daratumumab-bortezomib-dexamethasone (one case). Seven AL patients did not have specific treatment, due to the very advanced disease and the bad general performance status. ASCT was performed in seven cases at some point of the disease course in AL. In total, 22 patients were on doxycycline therapy among AL patients for at least three months. The only approved specific treatment for patients with wide type ATTR (ATTRwt) cardiomyopathy is tafamidis at a daily dose of 61 mg. It is not reimbursed in Hungary yet. Therefore, no patients with ATTRwt cardiomyopathy (CM) had tafamidis treatment. Six out of the eleven ATTRv patients had treatment with a daily dose of 20 mg tafamidis. One ATTRv and three ATTRwt patients participate in a double blinded, randomized clinical trial (HeliosB), where vutrisiran or placebo is given. Four patients were put on the waiting list for heart transplantation, and two of them were transplanted.

### 3.2. Clinical Characteristics of Patients with and without IWT

**Figure 1:**
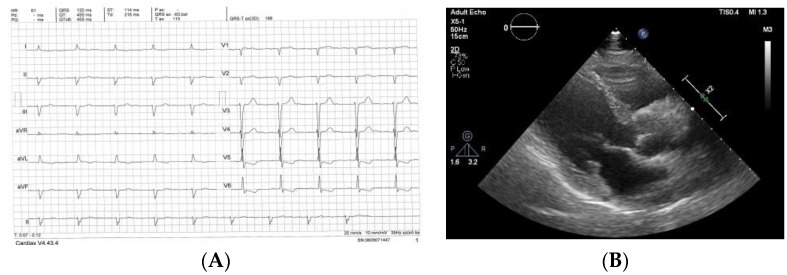

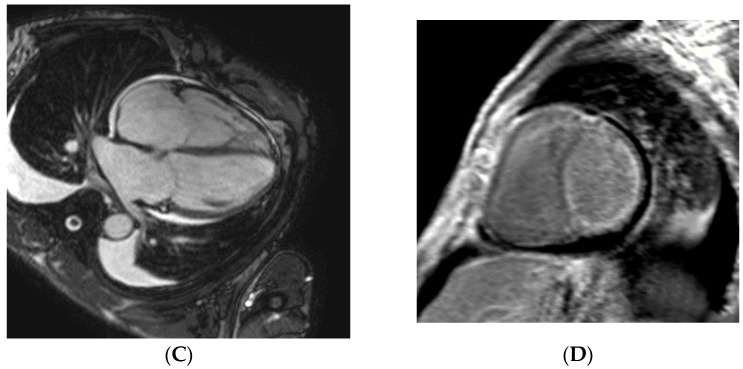
ECG, echocardiography and CMR images of patients without increased wall thickness and CA. (**A**): ECG: sinus rhythm, first degree AV block, no low voltage, ST-T changes. (**B**): Echocardiography. End diastolic frame, parasternal long axis view. (**C**): CMR image. Long axis four chamber view from a steady-state free precession movie sequence. End diastolic image. Normal left ventricular wall thickness and bilateral pleural effusion. (**D**): Late gadolinium enhancement from a phase sensitive inversion recovery (PSIR) sequence from the same patient in short axis view. Diffuse subendocardial enhancement, typical for CA.

We found 9 (9%) patients out of the 98, who had an average [(septal + posterior wall)/2] left ventricular wall thickness less than 12 mm. Typical CMR images and electrocardiogram (ECG) of a non-IWT patient are shown in Figure 1. A large proportion of the patients had advanced heart failure. There were only AL patients in the non-IWT group. All ATTR patients had IWT. All other parameters did not significantly differ between the two groups, but biomarker levels suggest that IWT patients had a more advanced disease than those with a normal mean wall thickness. These differences are not significant, but it is probably due to the sample size and the wide range and distribution of the measured serum levels.

In Table 2, we summarized the most important clinical, laboratory and echocardiographic characteristics of individual non-IWT CA patients. It is important to underline that all these patients had AL type CA. In four patients CMR showed typical signs of cardiac amyloidosis. It was based on the kinetics and morphology of the late enhancement of gadolinium-based contrast material. Mapping technics and calculation of extracellular volume was not considered diagnostic by itself for CA. One patient had diffuse late gadolinium enhancement (LGE) of the atria, suggesting CA on CMR images, but there was no sign of LGE in the ventricles. Two patients had biopsy-proven CA. In two patients CMR was not diagnostic for CA. In two patients CMR was not performed.

All non-IWT patients had AL; therefore, we compared clinical characteristics, imaging data and laboratory results between IWT and non-IWT AL patients, excluding other CA patients from the analysis, in order to avoid the bias caused by the presence of ATTR patients in the IWT group. The results are summarized in Table 3.

### 3.3. Prognosis of Patients with and without IWT

**Figure 2:**
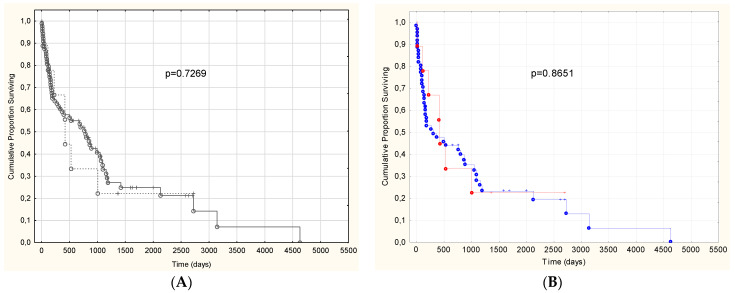
(**A**): Kaplan–Meier curves comparing cardiac amyloidosis patients with and without increased left ventricular wall thickness (IWT: *n* = 89, non IWT: 9; Log–Rank test: *p* = 0.7269 (**A**). (**B**): only AL patients (IWT: *n* = 61, non-IWT: = 9) are presented. There was also no significant difference in the outcome (Log-Rank test: *p* = 0.8651). Dash line represents non IWT patients, continuous line represents IWT patients.

When analyzing the outcome of the non-IWT group compared to IWT patients, we found no significant difference (Figure 2). We also performed the survival analysis among AL patients (with the exclusion of ATTR and the single AA patient) to avoid bias, that ATTR CA generally has better prognosis. The result was the same.

## 4. Discussion

In this retrospective analysis with the enrollment of consecutive CA patients, we validated the former observation [8] that a significant proportion of CA patients has normal left ventricular wall thickness. The novelty of our trial is the fact that we defined a lower cut off value for non-IWT (less than 12 mm), which is in accordance with the latest guidelines on CA [6]. We also described the clinical characteristics and clinical outcome of these patients.

For a long time, increased wall thickness was thought to be an essential morphological sign of CA. In clinical trials and guidelines, the presence of IWT was necessary for the diagnosis of CA [9,10]. Later, however, as the sensitivity of the diagnostic tools increased and the complication rate of endomyocardial biopsy (EMB) decreased, it became obvious that CA, especially AL type, is more common among patients with plasma cell dyscrasia and extracardiac evidence of systemic amyloidosis. In a consecutive series of 117 systemic AL patients between 1995 and 2012, it was found that 60% of them had cardiac involvement, and 25 patients had a mean left ventricular wall thickness equal to or less than 12 mm [8]. For the diagnosis of this latter group, they used EMB, CMR or a left ventricular wall thickness less than or equal to 12 mm with a low voltage ECG. The importance of the distinction between the definition of IWT (greater than 12 mm versus grater or equal to 12 mm) is nicely shown by the fact that in the abovementioned trial, 22 patients had a mean IWT of 12 mm out of the 25 non-IWT CA AL patients.

Later, in 2019, an expert consensus recommendation on the multimodality imaging in cardiac amyloidosis was published, endorsed by eight associations and societies from Europe and the USA, all involved in CA [4,5]. This recommendation recognized that both AL and ATTR amyloidosis are more common than it was thought, and the disease is underdiagnosed. It is of course partially due to the low diagnostic awareness, but also to the fact that the sensitivity of the imaging modalities is suboptimal. Therefore, this recommendation incorporated the use of cardiac biomarkers into the diagnostic arsenal. They recommended that AL CA can be diagnosed if an extracardiac biopsy proves the presence of systemic amyloidosis, CMR and echocardiography are not diagnostic for CA, but significant age-adjusted elevation in NTproBNP/BNP or troponin level is present, with the exclusion of all other causes for these biomarker changes. In our retrospective analysis, we used these criteria for the diagnosis of CA, in accordance with our local protocol and daily practice in our department. All these patients, including those who fulfilled these criteria but had no IWT, were treated for CA.

One may think that CA without IWT is a clinically insignificant form of this progressive disease, captured in a very early phase. Our data do not support this hypothesis because these patients also had a very significant elevation of NTproBNP and troponin levels. We also know that such an elevation is a poor prognostic sign for both AL and ATTR patients [11,12]. The fact, that the diagnosis of heart failure was prior to the diagnosis of CA and plasma cell dyscrasia (PCD) in four non-IWT patients, shows that these patients had clinically relevant cardiac symptoms. The presence of the so called “red flag” signs made it possible to find the systemic amyloidosis in the background of their heart failure. The other five patients were diagnosed first with PCD, and heart failure was found either as a screening for organ involvement or new onset heart failure symptoms were reported by the patients. In all nine non-IWT patients with clinical and histological evidence of AL, there was a significant elevation of NTproBNP. Five patients had positive CMR or EMB for CA, while four were diagnosed purely based on the biomarker elevation in addition to the extracardiac biopsy. None of them had other cause for NTproBNP elevation: no clinically relevant renal failure, hypertension, hypervolemia due to nephrotic syndrome, severe valve disease, atrial fibrillation with high ventricular response or other cause were present.

We found no significant difference between the outcome of the IWT and non-IWT group (Figure 2). In the survival analysis among AL patients (with the exclusion of ATTR who have generally better prognosis and the single AA patient), the result was the same. Lee et al. were also not able to show significant difference in survival between these groups [8], which suggests that non-IWT CA should be treated similarly as IWT CA.

In the light of our results, where all non-IWT patients had AL, we compared not only the outcome, but clinical characteristics, imaging data and laboratory results between IWT and non-IWT AL patients, excluding other CA patients from the analysis, in order to look for differences. The results are summarized in Table 3.

When comparing IWT and non-IWT group only among AL CA patients, we found, that cardiac biomarkers were higher in the IWT group, but the difference between the serum levels of the free light chains was almost the same. Interestingly, low voltage on ECG was significantly more common among IWT AL patients. Not shown in the table, but also interesting, is that apical sparing on strain analysis (according to the definition of Phelan et al. [7]) was not found in any non-IWT AL CA patient (test performed in 5/9 patients). Eight IWT patients had apical sparing among the twelve patients, where regional strain was examined. When diagnosis was clear due to prior CMR, positive cardiac biopsy or other fulfilled diagnostic criteria, the analysis was not performed.

In a recent study, Devesa et al. prospectively examined patients with heart failure and preserved ejection fraction (HFpEF) and no IWT, what they defined as a wall thickness less than 12 mm [13]. With the use of 99-Tc-DPD scintigraphy in 58 patients they found three patients with ATTR. This result shows that not only AL, but also ATTR CA, can be diagnosed without IWT. However, the clinical situation for the diagnosis of ATTR and AL CA is different. When systemic AL is diagnosed, screening for different organ involvement is performed, and it is also regularly performed in patients who are followed or treated for PCD [14]. It gives the opportunity to find CA in an earlier disease stage, and partially explains the fact that all of our CA patients without IWT had AL disease and not ATTR. The more common wild-type transthyretin amyloidosis (ATTRwt), as well as the hereditary form (ATTRv), is usually diagnosed when HFpEF and IWT is present [15]. AL has a significantly more diverse clinical picture than the common form of ATTR has. In ATTRwt, the carpal tunnel syndrome is usually the only clinically significant extracardiac abnormality [16,17]. On the other hand, in AL CA, peripheral polyneuropathy, nephrotic syndrome, skin lesions, gastrointestinal symptoms, symptoms and complains of multiple myeloma are often presented [17]. These symptoms may be the red flags, to think of the possibility of CA in HFpEF without IWT and without the previous diagnosis of PCD. We think that this is the explanation why we did not have any ATTR patients in the non-IWT group.

In a recently published multicentric trial [18], Boldrini et al. proposed the use of multiparametric echocardiographic scoring system for the diagnosis of CA. These scoring systems are different for patients with AL and patients in whom IWT is the first abnormal imaging finding. They focus on echocardiographic diagnosis of CA, and the proposed scoring systems incorporate strain analysis also, but no laboratory or clinical data. In this trial, the overall sensitivity of a septal wall thickness>12 mm was 84%, underlying the fact that there are patients with CA, and a relatively normal wall thickness.

The latest position statement of the European Society of Cardiology (ESC) on the diagnosis and treatment of CA [19] places IWT (equal to or thicker than 12 mm) to the center of clinical suspicion of CA. For ATTR CA bisphosphonate scintigraphy, as well as typical echocardiography/CMR, exams are needed for the diagnosis. However, echocardiographic criteria include a wall thickness higher than or equal to 12 mm. The document does not incorporate a possible echocardiography-based (non-cardiac biopsy, non CMR) diagnostic algorithm or diagnostic criteria for AL amyloidosis patients with biopsy proven extracardiac amyloidosis, but without IWT. It offers the diagnosis of cardiac involvement for AL patients with no IWT only if either CMR or EMB gives a positive, CA specific result. We think that this is in contrast with the result of the abovementioned trials and our findings. In many cases the use of EMB is avoided due to its invasive nature. CMR diagnosis of CA is based on the use of late gadolinium enhancement, but the use of gadolinium-based contrast material is relatively or absolutely contraindicated for patients with a GFR < 30 mL/min/1.73 m^2^, for patients with implanted devices, non MRI-conditional pacemakers, claustrophobia, orthopnoe and atrial fibrillation. The sensitivity of the LGE-based CMR is also less than perfect, around 80–83% in different studies, when the presence of global subendocardial or transmural LGE was used for the diagnosis [20,21]. Mapping sequences and calculation of the extracellular volume makes CMR more sensitive [22,23], but these methods are not part of the diagnostic criteria in this document. Therefore, the use of biomarkers to support the diagnostic process seems to be reasonable mainly for those with non-IWT CA.

Taken together: non-IWT CA is probably underdiagnosed. We think that diagnostic accuracy can be improved with regular NTproBNP screening of PCD patients and regular cardiac monitoring of asymptomatic TTR gene mutation carriers. It seems reasonable to screen non-IWT HFpEF patients for CA, especially if other causes of HF and elevated BNP (severe renal failure, fluid retention, severe anemia, clinically significant bradycardia or tachycardia, arterial-venous shunts) can be excluded. Screening of non-IWT HFpEF patients for the CA “red flags” may also improve the diagnostic accuracy for CA.

The main limitation of our study is that it was a single center trial. In recent years, there was a shift toward ATTR CA in the number among newly diagnosed CA patients [16], but this is not seen yet, due to the long time interval from where the patients were included. The retrospective nature of the study is not necessarily a limitation, because echocardiographer was not influenced when wall thickness was measured, not like in a prospective trial, focusing on CA and presence of IWT.

## 5. Conclusions

In this trial, we verified the former finding that there is a significant proportion of CA patients without IWT. We also validated that the prognosis of these patients is similarly poor as that of IWT patients. The novelty of our trial is the fact that we defined non-IWT as a mean left ventricular wall thickness less than 12 mm. Such a low cut-off value was not used previously in similar CA studies. We found that 9% of CA patients has no IWT. In our trial, all these patients had AL amyloidosis, and had a clinically significant CA and poor prognosis. In prior clinical trials and guidelines for the diagnosis of CA, normal wall thickness was defined as equal to or less than 12 mm. However, the latest ESC position statement defines normal wall thickness in CA as we did (<12 mm), and places IWT into the center of the diagnostic suspicion and diagnostic process of CA. We think that the diagnosis of non-IWT CA may be assisted by the use of NTproBNP and the search for CA “red flags” in non-IWT HFpEF patients when there is no other obvious cause of heart failure. 

## Figures and Tables

**Table 1:** Clinical characteristics, serum levels of cardiac biomarkers and the main echocardiographic parameters of the cardiac amyloidosis patients, grouped according to the presence or absence of increased wall thickness.

Characteristic	All Patients (*n* = 98)	Patients with IWT (*n* = 89)	Patients without IWT (*n* = 9)	*p* Value
Age (years)	68 (59–76)	68 (60–76)	59 (58–72)	0.281
Male patients (*n*, %)	54 (55)	50 (56)	4 (44.4)	0.500
NYHA III-IV (*n*, %)	51 (52)	47 (53)	4 (44.4)	0.574
ATTRv (*n*, %)	11 (11)	11 (12)	0 (0)	0.263
ATTRwt (*n*, %)	16 (16)	16 (18)	0 (0)	0.164
AL (*n*, %)	70 (71)	61 (68.5)	9 (100)	0.046
AA (*n*, %)	1 (1)	1 (1.1)	0 (0)	0.750
Mean wall thickness (mm)	16.5 (14–18)	17 (14.5–18.5)	10 (10–11)	**<0.001**
Septum (mm)	16 (14–19)	17 (14–19)	10 (10–11)	**<0.001**
Posterior wall (mm)	16 (14–18)	16 (15–18)	10 (10–11)	**<0.001**
Relative wall thickness	0.79 (0.65–1.02)	0.84 (0.68–1.02)	0.52 (0.43–0.55)	**<0.001**
Left ventricular ejection fraction (%)	54 (43–62)	54 (44–62)	56 (40–60)	0.725
E/e’(e’: average of lateral and septal e’)	19.5 (15.11–23.8)	20 (15.6–23.8)	15.82 (11.47–26.09)	0.298
TAPSE (mm)	15 (11–19.5)	16 (11–19)	19 (14–24)	0.119
NTproBNP (pg/mL)	4289 (1537–8575)	4819 (1713–8798)	1821 (573–4311)	0.172
Troponin T (ng/L)	73 (40–127)	83 (40–131)	50 (26–62)	0.090

Values are presented as medians with interquartile ranges (IQR), or as percentages. IWT: increased wall thickness, NYHA: New York Heart Association, ATTRwt: wild type transthyretin amyloidosis, ATTRv: variant transthyretin amyloidosis, AL: light chain amyloidosis, AA: amyloidosis secondary to inflammatory diseases, TAPSE: tricuspid annular plain systolic excursion, NTproBNP: N-terminal pro B-type natriuretic peptide.

**Table 2:** Clinical, laboratory and echocardiographic features of patients with cardiac amyloidosis, where the average of septal and inferior wall thickness was less than 12 mm. All patients had light chain amyloidosis.

Pt	Age at Diagnosis (Years) Sex of Patient	Left Ventricular Septal/Inferior/Average Wall Thickness (mm) EDV (mm)/ EF Measured with Echocardiography	E/e’	Stage of Heart Failure According to NYHA at the Time of Diagnosis	NTproBNP (pg/mL)	Troponin T (ng/L)	GFR (mL/min/ 1.73 m^2^)	Organ from where the Biopsy Proved AL Amyloidosis	Result of CMR	Known PCD Before the Diagnosis of CA	Complaint, Symptom that Led to the Diagnosis of CA/Other Clinically Significant Organ Involvement
1	65 male	10/10/10	33.5	IV	6492	50	23	rectum	typical for CA	No	heart failure/ polyneuropathy
		54/22									
2	59 male	10/10/10 43/56	8.95	II	2106	55	108	heart	typical for CA	Yes	heart failure/ polyneuropathy
3	52 male	11/11/11	16	III	1537	42	80	heart	typical for CA	No	heart failure/CTS, polyneuropathy
		42/63									
4	75 female	12/11/11.5 43/35	18.6	III	4311	96	112	skin of eyelid	NP	No	heart failure/-
5	59 female	10/10/10 36/63	14	I	1436	69	26	kidney	NP	Yes	screening for CA/ nephrotic syndrome
6	72 female	11/12/11.5 41/40	35.8	IV	6925	50	85	abdominal fat	typical for CA	No	heart failure
7	56 male	9/9/9 42/53	13.9	II	573	32.9	113	skin of eyelid, capsular ligament	LGE in atrial walls	Yes	screening for CA/ joint pain/ CTS
8	58 female	10/10/10 36/60	8.9	I	486	10	51	kidney	not typical for CA	Yes	screening for CA/ polyneuropathy/ nephrotic syndrome
9	82 female	11/11/11 51/60	15.6	II	1717	8	51	abdominal fat	not typical for CA	Yes	heart failure

Abbreviations: GFR: Glomerular filtration rate, NTproBNP: N-terminal pro B-type natriuretic peptide, CMR: Cardiac Magnetic Resonance imaging. NP: not performed, CA: cardiac amyloidosis, LGE: late gadolinium enhancement, PCD: plasma cell dyscrasia, EF: ejection fraction, Pt: patient. CTS: carpal tunnel syndrome.

**Table 3:** Clinical characteristics, serum levels of cardiac biomarkers and other laboratory parameters, and the main echocardiographic and ECG parameters of the AL CA patients, grouped according to the presence or absence of increased wall thickness.

Characteristic	AL Patients (*n* = 70)	AL IWT (*n* = 61)	AL non IWT (*n* = 9)	*p* Value
Age (years)	64 (58–73)	64 (58–73)	59 (58–72)	0.605
Male patients (*n*, %)	26 (37)	22 (36)	4 (44.4)	0.582
NYHA (III-IV) (*n*, %)	43 (30)	39 (64)	4 (44.4)	0.205
TroponinT (ng/L)	85 (45–132)	93 (46–141)	50 (26–62)	0.030
NTproBNP (pg/mL)	5063 (1909–11764)	5927 (2678–14183)	1821 (573–4311)	0.060
Mean left ventricular wall thickness (mm)	15 (13–17)	16 (14.5–17.5)	10 (10–11)	**<0.001**
Septum (mm)	15 (14–18)	16 (14–18)	10 (10–11)	**<0.001**
Posterior wall (mm)	15.5 (15–17)	16 (15–17)	10 (10–11)	**<0.001**
EF (%)	58 (43–63)	59 (45–63)	56 (40–60)	0.497
TAPSE (mm)	15 (11–19)	14 (10–19)	19 (14–24)	0.079
E/e’(e’: average of lateral and septal e’)	19.2 (15.6–25.5)	20 (17–25.5)	15.6 (14–18.6)	0.130
Low voltage on ECG (*n*, %)	39 (56)	37 (61)	2 (22)	0.032
Atrial fibrillation (*n*, %)	12 (17)	12 (20)	0 (0)	0.054
FLC-diff (mg/L)	227 (143–574)	224 (146–547)	376 (99–757)	0.666
GFR (mL/min/ 1.73 m^2^)	65 (44–81)	64 (44–79)	80 (51–108)	0.350
CyBorDex as first line specific medical therapy	33 (23)	28 (46)	5 (56)	0.459
ASCT	7 (10)	6 (10)	1 (11)	0.759

Values are presented as medians with interquartile ranges (IQR), or as percentages. AL: light chain amyloidosis, IWT: increased wall thickness, NTproBNP: N-terminal pro B-type natriuretic peptide. NYHA: New York Heart Association, EF: ejection fraction. TAPSE: tricuspid annular plane systolic excursion. FLC-diff: free light chain difference. GFR: glomerular filtration rate. CyBorDex: cycophosphamid, bortezomib, dexamethason. ASCT: autolog stemcell transplantation.

## Data Availability

Data is contained within the article.

## References

[1] Gertz M.A., Dispenzieri A. (2020). Systemic Amyloidosis Recognition, Prognosis, and Therapy: A Systematic Review. JAMA J. Am. Med. Assoc..

[2] Muchtar E., Buadi F.K., Dispenzieri A., Gertz M.A. (2016). Immunoglobulin Light-Chain Amyloidosis: From Basics to New Developments in Diagnosis, Prognosis and Therapy. Acta Haematol..

[3] Gertz M.A., Comenzo R., Falk R.H., Fermand J.P., Hazenberg B., Hawkins P.N., Merlini G., Moreau P., Ronco P., Sanchorawala V. (2005). Definition of organ involvement and treatment response in immunoglobulin light chain amyloidosis (AL): A consensus opinion from the 10th International Symposium on Amyloid and Amyloidosis, Tours, France, 18–22 April 2004. Am. J. Hematol..

[4] Dorbala S., Ando Y., Bokhari S., Dispenzieri A., Falk R.H., Ferrari V.A., Fontana M., Gheysens O., Gillmore J.D., Glaudemans A. (2019). ASNC/AHA/ASE/EANM/HFSA/ISA/SCMR/SNMMI expert consensus recommendations for multimodality imaging in cardiac amyloidosis: Part 1 of 2—Evidence base and standardized methods of imaging. J. Nucl. Cardiol..

[5] Dorbala S., Ando Y., Bokhari S., Dispenzieri A., Falk R.H., Ferrari V.A., Fontana M., Gheysens O., Gillmore J.D., Glaudemans A. (2020). ASNC/AHA/ASE/EANM/HFSA/ISA/SCMR/SNMMI expert consensus recommendations for multimodality imaging in cardiac amyloidosis: Part 2 of 2—Diagnostic criteria and appropriate utilization. J. Nucl. Cardiol..

[6] Garcia-Pavia P., Rapezzi C., Adler Y., Arad M., Basso C., Brucato A., Burazor I., Caforio A.L.P., Damy T., Eriksson U. (2021). Diagnosis and treatment of cardiac amyloidosis: A position statement of the ESC Working Group on Myocardial and Pericardial Diseases. Eur. Heart J..

[7] Phelan D., Collier P., Thavendiranathan P., Popovic Z., Hanna M., Plana J.C., Marwick T.H., Thomas J.D. (2012). Relative apical sparing of longitudinal strain using two-dimensional speckle-tracking echocardiography is both sensitive and specific for the diagnosis of cardiac amyloidosis. Heart.

[8] Lee G.Y., Kim K., Choi J.-O., Kim S.J., Kim J.-S., Choe Y.H., Grogan M.A., Jeon E.-S. (2014). Cardiac Amyloidosis Without Increased Left Ventricular Wall Thickness. Mayo Clin. Proc..

[9] Cueto-Garcia L., Reeder G.S., Kyle R.A., Wood D.L., Seward J.B., Naessens J., Offord K.P., Greipp P.R., Edwards W.D., Tajik A.J. (1985). Echocardiographic findings in systemic amyloidosis: Spectrum of cardiac involvement and relation to survival. J. Am. Coll. Cardiol..

[10] Bellavia D., Pellikka P.A., Al-Zahrani G.B., Abraham T.P., Dispenzieri A., Miyazaki C., Lacy M., Scott C., Oh J.K., Miller F.A. (2010). Independent Predictors of Survival in Primary Systemic (AL) Amyloidosis, Including Cardiac Biomarkers and Left Ventricular Strain Imaging: An Observational Cohort Study. J. Am. Soc. Echocardiogr..

[11] Kumar S., Dispenzieri A., Lacy M.Q., Hayman S.R., Buadi F.K., Colby C., Laumann K., Zeldenrust S.R., Leung N., Dingli D. (2012). Revised Prognostic Staging System for Light Chain Amyloidosis Incorporating Cardiac Biomarkers and Serum Free Light Chain Measurements. J. Clin. Oncol..

[12] Gillmore J.D., Damy T., FontAna M., Hutchinson M., Lachmann H.J., Martinez-Naharro A., Quarta C.C., Rezk T., Whelan C.J., Gonzalez-Lopez E. (2018). A new staging system for cardiac transthyretin amyloidosis. Eur. Heart J..

[13] Devesa A., Blasco A.C., Lázaro A.M.P., Askari E., Lapeña G., Talavera S.G., Urquía M.T., Olleros C.R., Tuñón J., Ibáñez B. (2021). Prevalence of transthyretin amyloidosis in patients with heart failure and no left ventricular hypertrophy. ESC Heart Fail..

[14] Ryšavá R. (2019). AL amyloidosis: Advances in diagnostics and treatment. Nephrol. Dial. Transplant..

[15] Razvi Y., Patel R.K., Fontana M., Gillmore J.D. (2021). Cardiac Amyloidosis: A Review of Current Imaging Techniques. Front. Cardiovasc. Med..

[16] Wechalekar A.D., Gillmore J.D., Hawkins P.N. (2016). Systemic amyloidosis. Lancet.

[17] Joury A., Gupta T., Krim S.R. (2021). Cardiac Amyloidosis: Presentations, Diagnostic Work-up and Collaborative Approach for Comprehensive Clinical Management. Curr. Probl. Cardiol..

[18] Boldrini M., Cappelli F., Chacko L., Restrepo-Cordoba M.A., Sainz A.L., Giannoni A., Aimo A., Baggiano A., Martinez-Naharro A., Whelan C. (2020). Multiparametric Echocardiography Scores for the Diagnosis of Cardiac Amyloidosis. JACC Cardiovasc. Imaging.

[19] Garcia-Pavia P., Rapezzi C., Adler Y., Arad M., Basso C., Brucato A., Burazor I., Caforio A.L., Damy T., Eriksson U. (2021). Diagnosis and treatment of cardiac amyloidosis. A position statement of the European Society of Cardiology W orking G roup on M yocardial and P ericardial D iseases. Eur. J. Heart Fail..

[20] Vogelsberg H., Mahrholdt H., Deluigi C.C., Yilmaz A., Kispert E.M., Greulich S., Klingel K., Kandolf R., Sechtem U. (2008). Cardiovascular Magnetic Resonance in Clinically Suspected Cardiac Amyloidosis: Noninvasive Imaging Compared to Endomyocardial Biopsy. J. Am. Coll. Cardiol..

[21] Syed I.S., Glockner J.F., Feng D., Araoz P.A., Martinez M.W., Edwards W.D., Gertz M.A., Dispenzieri A., Oh J.K., Bellavia D. (2010). Role of Cardiac Magnetic Resonance Imaging in the Detection of Cardiac Amyloidosis. JACC Cardiovasc. Imaging.

[22] Maceira A.M., Joshi J., Prasad S.K., Moon J., Perugini E., Harding I., Sheppard M., Poole-Wilson P.A., Hawkins P.N., Pennell D. (2005). Cardiovascular Magnetic Resonance in Cardiac Amyloidosis. Circulation.

[23] Messroghli D.R., Moon J.C., Ferreira V.M., Grosse-Wortmann L., He T., Kellman P., Mascherbauer J., Nezafat R., Salerno M., Schelbert E.B. (2017). Clinical recommendations for cardiovascular magnetic resonance mapping of T1, T2, T2* and extracellular volume: A consensus statement by the Society for Cardiovascular Magnetic Resonance (SCMR) endorsed by the European Association for Cardiovascular Imaging (EACVI). J. Cardiovasc. Magn. Reson..

